# Misinformation: an empirical study with scientists and communicators during the COVID-19 pandemic

**DOI:** 10.1136/bmjos-2021-100188

**Published:** 2021-11-25

**Authors:** Lisa Parker, Jennifer A Byrne, Micah Goldwater, Nick Enfield

**Affiliations:** 1 School of Pharmacy, Charles Perkins Centre, The University of Sydney, Sydney, New South Wales, Australia; 2 School of Medical Sciences, Faculty of Medicine and Health, The University of Sydney, Sydney, New South Wales, Australia; 3 Biobanking, NSW Health Pathology, Camperdown, New South Wales, Australia; 4 School of Psychology, The University of Sydney, Sydney, New South Wales, Australia; 5 Sydney Social Sciences and Humanities Advanced Research Centre, Faculty of Arts and Social Sciences, The University of Sydney, Sydney, New South Wales, Australia

**Keywords:** misinformation, science communication, qualitative research, meta research

## Abstract

**Objectives:**

To study the experiences and views within the health science community regarding the spread and prevention of science misinformation within and beyond the setting of the COVID-19 pandemic.

**Methods:**

An exploratory study with an empirical ethics approach using qualitative interviews with Australians who produce, communicate and study health science research.

**Results:**

Key elements that participants considered might facilitate misinformation included: the production of low-quality, fraudulent or biased science research; inadequate public access to high-quality research; insufficient public reading of high-quality research. Strategies to reduce or prevent misinformation could come from within the academic community, academic and lay media publishing systems, government funders and educators of the general public. Recommended solutions from within the scientific community included: rewarding research translation, encouraging standardised study design, increasing use of automated quality assessment tools, mandating study protocol registration, transparent peer review, facilitating wider use of open access and use of newer technologies to target public audiences. There was disagreement over whether preprints were part of the problem or part of the solution.

**Conclusions:**

There is concern from within the health science community about systemic failings that might facilitate the production and spread of false or misleading science information. We advocate for further research into ways to minimise the production and spread of misinformation about COVID-19 and other science crises in the future.

**Strengths and limitations of this study**

- Rich empirical data on views of science experts about misinformation at a time when the topic has high saliency in public and scientific communities.
- Includes information and discussion about newer, non-traditional science communication processes such as preprints and social media.
- Australian focus so may not have uncovered information more prominent in other jurisdictions.
- Limited by time and funds so did not aim for saturation and may have missed some relevant topics and concepts.

## Introduction

Public discussion of false or misleading information about COVID-19 has been a prominent feature of the current pandemic.^1 2^ Studies of Twitter and YouTube activity about COVID-19 in early 2020 showed alarmingly high rates of misinformation, with up to a quarter of tweets/popular YouTube videos containing false or unverifiable information.^3 4^ The spread of misinformation about COVID-19 has been described as a threat to public health^5^ since people with false beliefs about COVID-19 are more likely to act in ways that put themselves and global populations at risk.^6^ The spread of ‘misinformation’ on science topics - false, inaccurate or misleading information, with or without the intention to deceive - is not a new problem.^7–9^ It is, however, of particular concern during a pandemic because of the urgency of the situation and the need to rely heavily on each other to behave responsibly.

Some notable cases of misinformation spread occurred during the early stages of the global pandemic. In January 2020, Pradhan and colleagues posted a paper on the bioRxiv preprint server about the molecular structure of SARS-CoV-2, suggesting the virus was ‘uncannily similar’ to HIV and unlikely to have evolved naturally.^10^ Although the study was criticised by the scientific community^11 12^ and retracted by the authors after just 3 days, it nevertheless spread widely, achieving the highest Altmetric Attention Score ever at the time, and sparking a global conspiracy about Chinese bioweapons. In another example, authors of a small observational case series in France,^13^ with questionable methodological rigour,^12^ reported a significant reduction in SARS-CoV-2 viral load in 20 patients treated with hydroxychloroquine versus 16 patients given usual care. The study was released on YouTube on 16 March,^14^ published in a peer-reviewed journal on 20 March^13^ and tweeted by Donald Trump on 22 March. Subsequently, there were chloroquine drug shortages, restrictions on outpatient dispensing, new legislation around prescribing, self-poisonings and deaths.^15–17^ In May 2020, a very large, multicentre observational study of chloroquine treatment in patients hospitalised with COVID-19 was published in the *Lancet*, showing that chloroquine was of no benefit.^18^ In response to the publication, large clinical trials of chloroquine treatment were immediately halted. It was subsequently alleged that the study was based on fraudulent data,^19^ the publication has been retracted and trials recommenced.^20^ It seems likely that these and other examples of false or misleading science research have become prominent during the COVID-19 pandemic because of the enormous public interest in science news and the huge stress on the science research system to produce new information at speed.^21^

The existing literature on the rise and spread of false or misleading science suggests several causes and debate over solutions. Flawed research can be due to sloppiness, bias or deliberate fraud, manifesting in ways such as low-quality methodology, misleading spin of results or falsified data.^22–24^ The spread of flawed research has also been attributed to systemic problems within science such as commercial influence^25^ and competitive pressure within academic communities to ‘publish or perish’.^26^ Even if science information is later shown to be false, the well-recognised phenomenon of causal imprinting means that it is hard for us to get rid of old ideas or unlearn fake news.^27^ The science community uses a range of tools to minimise the production and spread of flawed research, including: research ethics training and oversight,^28^ risk of bias tools,^29^ funding transparency,^30^ requirements for pre-registration of clinical trials,^31^ monitoring of research integrity,^23^ peer review of scientific publications^32^ and professional education about cognitive biases.^33^ Some science researchers have advocated for much wider uptake of quality assessment tools in academic publishing houses.^11 34 35^ Others have identified flaws in academic peer review and introduced more transparency^36^ or advocated for preprint servers to facilitate the rapid, freely available spread of science information.^37 38^ There is also a substantial literature focused on the target audiences, exploring how and why people believe false or misleading information.^39–41^ Recent studies have identified that particular subgroups are more likely to agree with misinformation about COVID-19 (eg, younger age, male gender, lower education, lower numeracy skills) and have recommended that public health authorities make particular efforts to target those audiences.^6 42^

There have been numerous news reports, Twitter conversations and opinion articles on the topic of misinformation in science and some more recent studies on problems associated with misinformation among the ‘infodemic’ of COVID-19 science.^1^ A 2020 survey of medical and academic experts showed concerns about possible facilitators of misinformation (eg, social media, television, scientific authors, academic publishing systems, target audience issues) and enthusiasm for solutions such as more stringent and automated journal screening of manuscript submissions.^43^ We aimed to add to this pool of data on experts’ attitudes about misinformation during the COVID-19 pandemic through an in-depth study of the collective experiences and views of those involved in producing, spreading and studying biomedical science research to obtain real-world insight into the processes involved and to identify current views on how to reduce misinformation. Ultimately, we want to inform conversations about how to facilitate timely spread of high-quality science while protecting the public from misinformation arising from the spread of low-quality science.

Our research goals were:

- To identify and analyse the views of science knowledge experts about the spread of science misinformation using the context of the COVID-19 pandemic as a key case study and stimulus for discussion.
- To identify strategies to reduce spread of scientific misinformation into the future.

## Methods

We used an empirical ethics case study approach incorporating qualitative research. Empirical ethics is a growing discipline that combines empirical research with theoretical reflection on a topic of ethical importance, aiming to deliver normative guidance.^44^ Our empirical work employed qualitative interview methods informed by well-established traditions, particularly constructed grounded theory as practised by Charmaz, although this was not a pure grounded theory study.^45 46^ Qualitative research methods are well suited to identifying processes and understanding people’s views. Our methods are reported in keeping with the Consolidated Criteria for Reporting Qualitative Research guidelines.^47^

### Author experience and reflexivity

The authorship team included researchers with a variety of professional backgrounds including clinical medicine, ethics in health and research, molecular biology, psychology and linguistics. All authors had research interests in scientific misinformation as well as research experience in qualitative research methods (LP), commercial influences in health (LP), publication ethics (JAB), cognitive bias (MG) and linguistic and cultural transmission of information (NE).

### Sampling and recruitment

We used Australia as our case study. We conducted purposive sampling to recruit participants with a range of experiences in the practice, spread and receipt of new scientific knowledge relating to health. Since we aimed to hear a wide range of experiences and views regarding dissemination of scientific information, we sought participant diversity in expertise, experience and roles. We sampled from early/mid-career researchers and more experienced academics working in science research or meta-research (studying issues of health research quality and integrity). We also sampled from science communicators working in academic publishing, university media offices or lay media outlets; and members of the lay community. This paper reports on the subsection of interview data from participants who were professionally involved in science knowledge production and communication.

We identified potential participants for interview through: existing professional and lay contacts; following up suggestions from previous participants (purposive snowball sampling^48^), searching for people currently publishing in the public domain about scientific misinformation in the field of COVID-19 pandemic. We contacted potential participants through our professional email networks and individual contacts, and through contact details in the public domain.

This was planned as an exploratory study. We were constrained by time, resources and the incentive to complete our research in a timely fashion in order to contribute useful information that might improve the process of science communication during the current pandemic crisis. We did not aim for data saturation, rather we aimed to recruit at least two participants from each of the major subgroups of: science researcher, meta-researcher, science communicator and lay community. In this way, we hoped to collect a broad range of information about the views and experiences of science professionals on the topic of misinformation in the early months of the COVID-19 pandemic.

### Data collection

LP obtained consent from participants to conduct semistructured interviews which were recorded and professionally transcribed. Interviews were conducted between May and July 2020. She introduced herself as a medical clinician with research interest and experience in research integrity. She asked about participant experiences with science research and/or science communication, and their views about flawed research and misinformation in the context of COVID-19 and more generally. Interviews were digitally recorded and professionally transcribed. Transcripts were deidentified.

### Data analysis

LP wrote field notes after all interviews. Interviews were discussed at regular group meetings and three transcripts were shared among the team. These shared transcripts were selected for diversity of participant characteristics and originality of conceptual data. After the first five interviews, LP drew up a preliminary list of codes that captured salient topics, informed by group discussions, prior reading and by the early data. LP coded the transcripts using NVivo software. She reviewed the list of codes as new concepts emerged in later interviews and from ongoing group meetings, and previously coded transcripts were recoded. LP conducted preliminary analyses, drawing on relevant literature in research and public health ethics.^49–51^ She wrote memos on emerging themes and shared these at group meetings, where analytical ideas were discussed and refined. We combined empirical work concurrently with analysis such that each could inform the other. For example, early codes included publication bias and academic pressure to publish as possible facilitators of misinformation, and preprints as potentially problematic science communication pathways. This early data prompted us to probe more deeply into these problems when talking with meta-researchers about possible strategies to reduce the production of low-quality research, while remaining open to new concepts and concerns. As data collection progressed, we used participant comments along with our existing knowledge to flesh out a conceptual map of contemporary processes of science research creation and communication and used that map as a scaffold for thinking about the data. Specifically, we considered locations within those pathways of knowledge creation and dissemination where systemic problems might sit that could facilitate misinformation, and where solutions might be effectively targeted.

### Ethics approval

We committed to protecting participant confidentiality and obtained consent from participants based on that commitment. Interview transcripts are not available for public reading because there is no true way to fully deidentify the entire transcripts, which contain contextual information and detail that mean participant identity could be exposed, particularly to other people who work in their field. We have provided selected, deidentified quotations within the paper, taking care to select quotes that make it unlikely for participant identities to be inferred.

## Results

We interviewed 16 participants, 10 women and 6 men, from a range of backgrounds as per table 1. The interviews were conducted over Zoom or telephone and lasted a mean of 59 (range 44–80) min.

**Table 1:** Characteristics of participants

Participant expertise/experience	Invited and participated n=16 (10 women)	Invited but did NOT participate n=12 (6 women)*
Early/mid-career researcher	3 (3)	2 (1)
Experienced researcher	6 (2)	4 (2)
Science communicator	5 (4)	5 (3)
Citizen	2 (1)	1 (1)

*Reasons included: did not respond to email (7), refused (4) and email failure (1).

Participants were all aware of the issue of misinformation in relation to the current pandemic. P1 (science communicator) described how misinformation had become a well-recognised topic within the lay press, such that it was typically a story in itself in relation to important events:

From the beginning I knew that the misinformation conspiracy theory … would be a thread flow all the way through … Any sort of momentous public occasion is now accompanied by spread of misinformation online. (P1, science communicator)

At the same time, however, participants identified that the spread of misinformation had become particularly intense during the pandemic. They talked about the newness and urgency of the situation leading to intense professional and public interest in new scientific knowledge.

### Processes of communicating scientific knowledge during the COVID-19 pandemic

**Figure 1:**
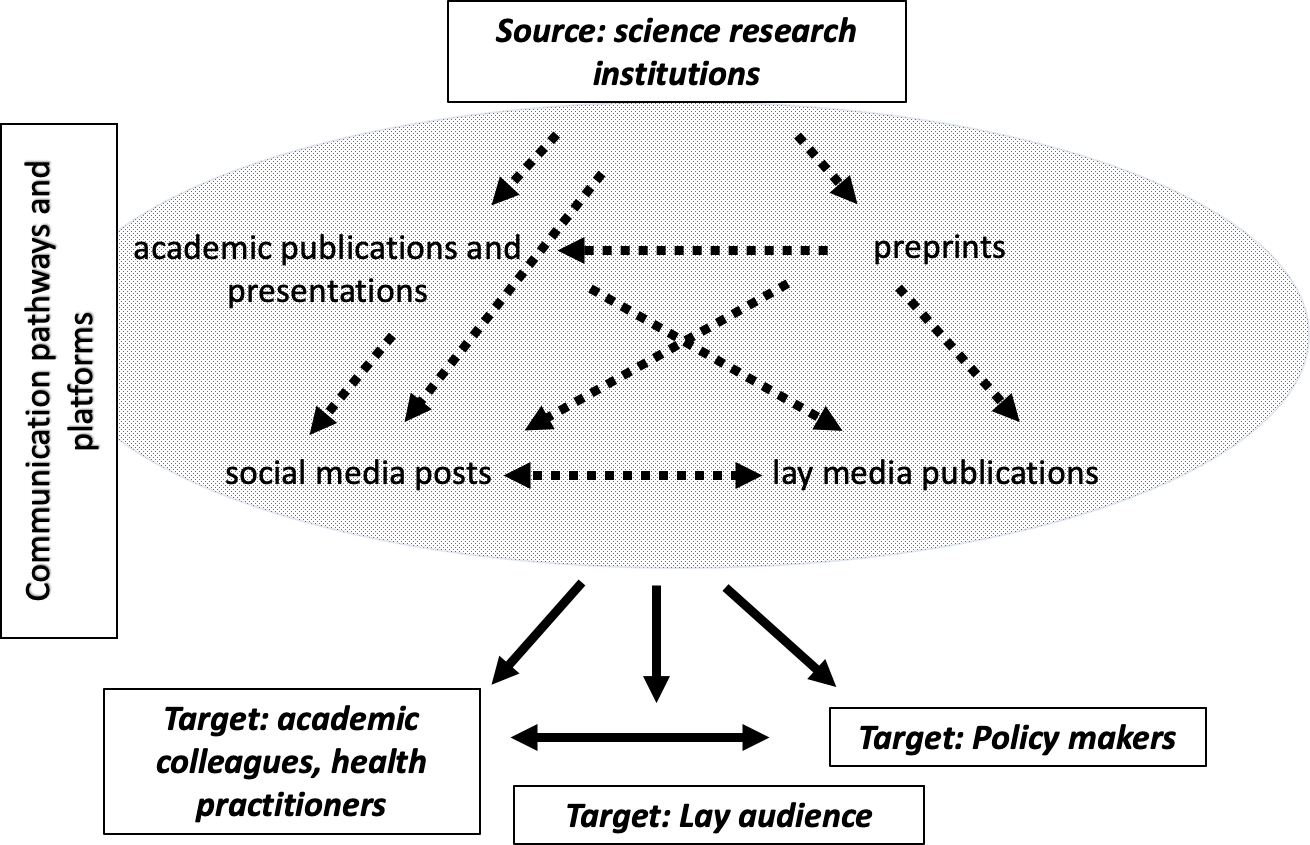
Participant experiences and views on the processes and interplay of factors for generation and communication of science during the COVID-19 pandemic, from source to target audiences. We drew on our background knowledge of science communication and empirical data from our interviews to sketch out pathways of science communication. This diagram is simplified for readability. According to participants, the flow of information was not necessarily linear and we have not included all the possible overlaps and influences.

We drew on participant descriptions of science production and communication to identify a range of processes through which new science research about COVID-19 was likely to have been communicated to the lay public. We present this as a process whereby information flows through a series of steps or ‘filters’ (see figure 1 for a variety of possible communication pathways and platforms and box 1 for a description of these knowledge dissemination processes). Ideally filters reject poor-quality, inappropriate or off-target information while facilitating the spread of good quality information in a form that is understandable and engaging for the target audience. This process model assisted our analysis of participants’ conceptualisation of how and why misinformation could result. The model also helped to analyse participant comments on where to direct preventive measures to minimise the spread of misinformation.

Box 1 **Participant-informed description of traditional and new process of science knowledge production and spread.**

The traditional process of science information production and spread was described by participants essentially as follows: the science research community generates ideas, obtains funding and conducts empirical research. The communication of that research must successfully pass through a series of ‘filters’ in order to reach the lay audience. These filters are assumed to remove all the low-quality science and let through all the high-quality science, although participants said that filter systems could potentially remove high-quality science as well, such as studies that were not thought to be of sufficient interest to the intended audience. Filters also assist with translation into language and concepts that are suitable for the target readership. In order to traverse this traditional filter system, researchers first submit their completed study manuscripts to academic publishing houses and/or conference organisers for communication to their academic colleagues. Many submissions are rejected outright but some submissions progress to in-house review and largely unpaid peer reviewers working in a similar field. Many reviewed studies are subsequently rejected but some are published in academic journals, often only available to academic colleagues via institutional library subscriptions. Some academic publications are read by science journalists and translated into news articles in the lay media. This might be facilitated by a university media office press release. The resulting news might be read, understood and believed by general readers and policymakers—or not.

Many participants noted that recent changes allow researchers to bypass one or more of the filters. For example, science researchers might choose to release their academic publications directly to academic peers and the public via online preprint servers, before or instead of submitting their work for conference presentation or academic publication. The science research community might use social media to publicise their journal articles and conference presentations to colleagues, the lay media and the general public. Participants were aware that the process might be affected by the addition of spurious or ‘junk’ science, that is not produced through the scientific research community, but which might enter the science communication system through the lay media and/or directly to the lay reader through social media postings and sharings.

### Key findings

Participant views on causes of misinformation collected predominantly around particular points along the entire pathway from science production > science communication > target audience access and interpretation. Participants considered that poor science research and communication practices could potentially enable the production of bad science, leaving the public with inadequate access to high-quality science information. Participants also spoke about what they perceived as a lack of attention to educating the public about how to access, identify and interpret reliable scientific information.

We identified dominant views among participants on how to reduce or prevent misinformation. Participants suggested that key changes could come from within the academic community, academic and lay media publishing systems and government funding processes. There was disagreement over whether preprints were part of the problem or part of the solution. Upskilling the lay public in science and information literacy was seen as an important adjunct to changes within the science and science communication communities. The key problematic elements and participants’ related solutions are presented in table 2 and discussed in more detail in the following text. Most participants spoke about multiple causes of misinformation, but typically focused on one key element as being the dominant problem. We identified limited associations between participant experience and the key element that dominated their views. For example, ‘Inadequate Access’ was the main issue discussed by five of the six science communicators but only by two of the nine researchers (early/mid-career and more experienced). ‘Bad Science’ was the main issue discussed by five of the researchers and none of the science communicators.

**Table 2:** Participant views on reasons for the prevalence of misinformation and possible strategies for change

Reason for the problem	Example quotations	Strategies for change
**Bad science**—poor-quality or biased science research		
Academics experience huge pressure to publish.	‘We have pressure to publish for publications and for grants and also in terms of promotions … I [think], not only governments but funding sources, they should not put that much pressure on how many papers we publish, but in the quality.’ (P2, early/mid-career researcher)	Change the incentives for researchers—reward translation activities rather than just publication numbers.
Commercial influences.	‘If you have a study [that] is funded by industry, you’re going to get cited much more often and you’re also going to get cited much more quickly in systematic reviews. And so what happens, overall, is that systematic reviews now end up reflecting industry funded studies much more heavily … One of the things that I think is really useful for when we're talking about primary research … is around synthesis ability … checklists are really useful for that. Just to say, “These are the things that you need to think about when you’re designing and then reporting your study that are going to make it easier for us to do our jobs in evidence synthesis”.’ (P3, experienced researcher)	Increase public funding of science to minimise commercial influences; train researchers in synthesis ability.
Questionable research practices, for example, recruiting until results are statistically significant then stopping.	‘You see a lot of people doing very poor statistics …[I’ve seen] multiple papers in [high impact factor journals] that are terrible. It’s not just the poor journals … I think in the future we’re going to be more heavily scrutinised… We could look at your p-values and if your p-values are always just below.05 you might need to explain yourself.’ (P4, experienced researcher)	Increase oversight of research quality in academic institutions and publishing houses.
**Inadequate access**—lack of access to research that is free, timely, understandable and trustworthy		
Publication bias: for example, only publishing results that are favourable to funders/political leaders.	‘Share a league table of people who publish their protocol, whose protocol matched the actual analysis … to reward the good behaviour, to get the big institutions and the big funders in government to prioritise that and put that on a pedestal. Maybe that could be part of the block funding, how well you’re doing that. And then straight away, that dramatically changes the incentive, and then everybody has to follow suit on that.’ (P4, experienced researcher)	Encourage protocol registration.
Impenetrable language, concepts and loss of specialist science journalists who can explain and critically evaluate scientific studies.	‘There needs to be a middle ground between the press release which is, “This is going to cure cancer” and the scientific paper, which is impregnable … Maybe just like a one-pager that’s like, “This is what we’re seeking to find out. This is a preprint. This is how many people. These are the shortcomings…and this is where this research fits into the arc of research” …It’d certainly be good for health journalists. Because not every outlet has dedicated health reporters anymore anyway”.’ (P1, science communicator)	Reward plain language publications, including simplified versions published in tandem with full studies.
Peer review system is inefficient, lacks transparency.	‘A colleague told me a ridiculous story where she was sent a paper … for rapid peer review for COVID, and she reviewed it … within a couple of days and said, “This is terrible,” and … they’d actually published it by then.’ (P4, experienced researcher)	Open peer review, with academic reward.
Academic publication paywalls.	‘We’re a really unusual journal in that we’re completely open access. But … it’s certainly not a viable business model. I don’t know how much longer we can do it for.’ (P5, science communicator)	Open access facilitated by governments, funders and institutions.
**Low information and science literacy**—people do not read high-quality science information		
People use unreliable and algorithm-driven sources for science news.	‘[Don’t] read all your news on Facebook, you have to read something else! … [Because] social media … are so good with these algorithms, you’re only going to see what’s going to reinforce what you already think.’ (P6, early/mid-career researcher)	Educate the public about where to find and how to evaluate good science.
People are less attentive to trustworthiness of news than its visual or narrative appeal traditional.	‘I think there’s this disconnect between what goes on in the health research world and the findings and then what all the, especially younger people are looking at on the internet … I look at people when they’re looking at their mobile phones and they just flick through so quickly. So the amount of time you have to get someone’s attention is so miniscule now … It’s almost incidental that you’re impressing upon them that this is a trusted source of information because I think a lot of people don’t even ask that question.’ (P7, science communicator)	Train scientists to use engaging communication tools: visuals and narrative.
People expect certainty, precision and immediate answers in science.	‘Science is about embracing uncertainty, actually. That’s where it is strongest, I suppose. That’s what it is. Whereas I think the popular imagination is scientists can deliver certainty. Scientists know things. And I think it’s partly the way it’s taught in schools. Mathematics and science, you know? There is a correct answer.’ (P8, science communicator)	Educate the public about the scientific process.

### Bad science

Don’t believe what you read in a journal. Just because it’s in a journal it doesn’t mean it’s true. (P6, early/mid-career researcher)

Many participants talked at length about the production of low-quality, fraudulent or biased science as a cause of misinformation. Within their comments we identified three main ideas about triggers or facilitators for this kind of ‘bad science’: institutional pressure to publish, high competition for academic science jobs, inadequate training in research misconduct. More broadly, participants talked about systemic bias resulting from widespread industry funding, whereby meta-analyses were more likely to include industry-funded primary studies because these tended to be reported in a standardised way, facilitating easy synthesis ability (see table 2).

A dominant view among participants was that ‘bad science’ was not specifically a COVID-19 phenomenon. P4 (experienced researcher), for example, expressed astonishment that the scientific community was surprised by COVID-19-related research fraud:

I’m shocked that these two top scientists are shocked [about the retracted and allegedly fraudulent Surgisphere study]. This has been happening repeatedly, over and over again, before this crisis. (P4, experienced researcher)

We identified several themes within participants’ comments about how to reduce the production and communication of bad science. A strong view, particularly among science researchers, was that the scientific research system should change to reduce the pressure on researchers to publish. Academic institutions and granting bodies should concentrate on rewarding translation activities rather than citation rates or publication in high-impact journals. At the same time, there was a call for greater use of checklists to assist researchers, including smaller groups relying on public funding, design studies that could be easily synthesised into meta-analyses.

Other participants saw low-quality research as being so prevalent that they focused more on preventing publication. For example, P3 (experienced researcher) saw important strategies to reduce misinformation from bad science could come from within academic publishing houses:

I see no reason why [journal publishing houses] aren’t already trying to do that for things like impossible p-values or duplication of images or photoshopping of western blots or whatever it is that people are manipulating images for at the moment. I mean there are tools to automatically detect image manipulation. I don't see why journals wouldn't be already using them. (P3, experienced researcher)

Wider use of in-house statistical review and plagiarism/image duplication detection tools could help academic publishers to detect and reject sloppy or fraudulent research submissions.

### Inadequate access to high-quality research

Open access is important because there are people that don’t have access to the information that they need because it’s behind a paywall. These are people that might be writing policy!(P9, experienced researcher)

Many participants said that elements within processes of science communication between researcher and the public meant that the public had inadequate access to high-quality information. For example, one participant spoke about a completed research study they’d been involved in that the funder insisted not be published, because the results were commercially and politically unfavourable to the funder. Others talked about how research papers might still be inaccessible even when published, since they were often written in a style that was impenetrable to readers outside the immediate field of study. There were many criticisms of academic publishing systems. There was concern that journal editors interested in impact factors tended to selectively publish studies likely to have high readership, typically positive findings and topical issues, meaning that important but less immediately topical studies might not find a publication outlet. The publication process was characterised as unacceptably slow, delaying the communication of research. Participants were critical of the peer review system, which typically relied on unnamed academics whose own biases and expertise were unknown to the reader. Importantly, participants were disapproving of academic publishing paywalls which effectively blocked access to science articles for the majority of the population who did not have an academic institutional affiliation. The lay media was also noted to be subject to editorial influence and selection bias, reporting preferentially on science news thought to be of immediate interest to readers and palatable to political allies and commercial sponsors. There was insufficient public funding for ‘public interest’ journalism that might be less likely to be subject to commercial bias.

Suggested strategies for change were multipronged. Participants called for academic institutions to mandate pre-registration of trial protocols to prevent post hoc selective data analysis aimed at producing results favouring the funder. They spoke about institutions encouraging researchers to write in plain language, and to produce simplified summaries of their findings (avoiding spin), that lay media journalists could draw on for communicating complicated ideas and findings. We identified several suggestions for change within peer review. For example, some participants called for peer review to be better rewarded in order to encourage academics to invest more of their time and effort in this important work. Some participants also advocated for open peer review, suggesting that reviews and reviewer identities should be publicly available so that readers could readily assess the background and expertise of reviewers. Other participants promoted the expanded use of preprint servers, which they regarded as facilitating transparency, more timely release of information and free access. Open access to academic papers was widely viewed as important and one participant commented approvingly of government initiatives in Austria working towards country-wide open access to academic publications.^52^

### The lay public do not read high-quality science information

People in school need to be taught that the internet is like reading graffiti on the wall. I mean you don’t believe what you read when someone writes graffiti on the bathroom stall. (P10, experienced researcher)

Participants also focused on the lay public audience when considering process problems that might lead to science misinformation. There was concern that members of the public might see and read more low-quality science information than high-quality science, and that they were not necessarily aware or concerned about this. Individuals might have poor information literacy, relying on algorithm-driven news feeds on social media sites, and not realise that this meant their news was likely to be skewed towards particular opinions or viewpoints. Many members of the lay public might be unable to judge the reliability of a news item or news source or might be interested in news as much for its entertainment value as for its informative value, and so not mind about the reliability of the content. Even people who source, read and value high-quality science information might have low science literacy: they might not understand the significance of individual studies, or the expected level of uncertainty within scientific research.

Participants discussed strategies that might address low levels of information or science literacy, recommending enhanced school and university education in those subjects. Others suggested that researchers embrace different communication style platforms such as infographics, which provide engaging visuals along with short, understandable content.

### Levers for change

Among participant views on how to manage the problem of science misinformation, we identified four dominant change agents (see table 3). Participants suggested policy and practice changes that could be introduced by relevant organisations. Some recommendations were detailed, focused and specific (eg, increased marks on university assignments for visual presentation and general appeal to improve science communication skills among graduates) while others were broader and more aspirational (eg, increased school-based education to improve information and science literacy). Several ideas relied on increased funding, but there were no comments about where the money might come from.

**Table 3:** Participant suggestions on policy and practice changes to reduce the spread of low-quality science

Change agents	Recommended actions
Scientific research institutions	Increased education about research integrity, for example, university assignments with research integrity content to ensure students must engage with the topic. Increased university teaching for science undergraduates on how to produce more visually engaging science communications; substantive marks in assignments for presentation and appeal; penalties for obscure jargon and acronyms. Rewards for publication in relevant (rather than high-impact) journals and translational activities such as engagement with community agencies. Enforcement of protocol pre-registration. Encouraged use of checklists to facilitate standardised study designs and reports that can be easily synthesised into systematic reviews. Recognition for peer review activities and/or mandated number of open access peer reviews.
Academic publishing systems	Mandated open peer review to allow readers to assess rigour. Mandated use of automated surveillance tools to look for bias and fraud, for example, cross-checking and tracking author’s conflict of interest statements and industry funding statements across different publications. Increased use of in-house expert statisticians for statistical scrutiny of submitted manuscripts. Improved postpublication review processes to enable more timely error correction and/or retraction of flawed research.
Public funding agencies	Government-funded country-wide open access to academic journals.^80^ Increased public funding for science research to create better job security for researchers and help reduce the ‘publish or perish’ culture. Increased public funding for news media to ensure different (non-commercial) voices are heard.
Educators	Increased school and university-based education on where to find high-quality information sources, how to assess trustworthiness of sources, limitations of algorithm-driven news feeds. Increased school education in science experimentation to encourage better understanding of key concepts such as uncertainty and reproducibility.

## Discussion

### Principal findings

In general, we found that participants were cognisant of the spread of science misinformation. They recognised the increased salience of this issue during the current COVID-19 pandemic but considered it to be a long-standing problem. Participants considered the cause of science misinformation to be multifactorial. Our results show they located possible triggers or facilitators of misinformation at one or more key points along the pathway from science research production through to communication and target audience access and interpretation. In particular, problems were seen as sitting within the broad domains of production of ‘bad’ or poor-quality science; inadequate communication practices, meaning insufficient public access to trustworthy science research; and lack of attention to sparking public interest and understanding about science.

### What this study adds and correlation with existing literature

Our findings indicate that scientists are not satisfied with the current quality of science research and remain deeply worried about ‘bad’ science. These kinds of concerns persist despite many years of awareness about systemic problems, which one might have hoped would have improved triggered interventions that achieved confidence in research quality.^8 26 53^ The science and science communication communities contain people who are strongly critical of their own processes for generating and spreading science information and accepting of at least partial responsibility for the problem of misinformation. Participants’ acknowledgement of ongoing systemic failures within the science research community resonates with the literature.^7 23 29 54^ Participants’ criticisms of science communication processes have also been discussed in the literature: for example, many scientific studies are unavailable because of academic journal paywalls^55^ or because commercial or political pressures ensure they remain unpublished.^56^ Published papers often contain errors.^57–59^ Inappropriate analysis, spin and fraud are persistent problems.^60–62^ An important finding from our data was that, despite published criticisms about preprints,^63^ many of our participants were in favour of this new publication process. Participants recognised that the public might not currently understand the implications of a non-peer-reviewed preprint, but were confident that public education could manage this and overall saw open access to science and peer review as part of the solution, rather than part of the problem.

Our results echo the literature that suggests science communities tend not to engage heavily with the issues of research misconduct and fraud.^23^ Participants did acknowledge systemic pressures that might lead to research misconduct, but there was limited discussion about problematic individuals or the need for solutions to research fraud, despite this being an ongoing problem in science.^62 64–66^ Our interviews were conducted after the retraction of the Uncanny Similarities preprint; participants who referred to that study generally dismissed it as opportunistic spin rather than falsification of data. Most of our interviews were completed before the Surgisphere scandal and publication retraction,^20^ which was widely described in the media as deliberate fraud, so it is possible that falsified data and deliberate fraud would have come out more strongly if the timing of interviews had been different. However, given the importance and seeming frequency of fraud, we are concerned that this problem is not more widely acknowledged among the science and science communication communities, and we would advocate for broader public discussion about this. Some are concerned that public awareness about fraud might reduce public trust in science further,^67 68^ but surely it is unreasonable to expect the public to trust a community that cannot acknowledge and will not look to fix its own ongoing problems.

Our study showed that participants were also cognisant of problems that are largely external to their own communities. This includes the problem of epistemic bubbles, whereby target audiences lack exposure to good science because of reliance on poor-quality or algorithm-based sources,^69 70^ and communities that are disengaged from science or science communicators because of reasons such as disinterest and cognitive bias.^71 72^ In the main participants tended to consolidate their views around the deficit model of science communication, viewing the causes and solutions of misinformation predominantly through an assumption that the fundamental problem is lack of public knowledge and/or understanding about science.^73 74^ We heard only minimal comment about the importance of understanding what might affect people’s beliefs and behaviour other than successful communication of high-quality science information, such as their social identity and moral values.^75^ This may reflect the professional focus of our expert participants and may also align with the biases of the authors, who collectively shared professional experience and interests with the participants. An ongoing challenge is to distinguish between (1) how people access and process (potentially limited, biased) information in decision-making and (2) people’s personal beliefs and meta-theories about how (1) works. We have interviewed science experts, who will by nature contribute elements of (1) and (2)—future research will need to explore the actual relation between the two. For example, to improve scientific communication we need to understand the degree to which expert science communicators act as if people solely form beliefs based on the quality of information they are exposed to (rather than effects of social identity and moral values) and the degree to which people’s beliefs are actually formed in this manner.

### Strengths and limitations of this study

The novelty and originality of this study lie in the rich detail of the data set across a range of science experts at this important point in time when there is widespread concern about the impact of misinformation on the progress of a global pandemic and simultaneously, growing awareness and accelerated use of science communication strategies such as Twitter and academic preprint servers in the health science sector. Given this timing, science professionals are likely to have been thinking and reading about the problems and need for solutions with regard to misinformation, providing useful reflections for analytical review. This study provides a holistic, connected view of the complexities within the system. At the same time, this study had limitations. We concentrated on the Australian context so we might not have had access to political or systemic issues that might be more prominent in other jurisdictions. We were limited by timing and resources so did not aim for saturation, meaning that some topics and concepts were not explored. For example, the problem of predatory journals that might contribute to misinformation by offering soft peer review was not discussed although it is well known among academic communities.^76^ Finally, this paper focuses on experiences and views within the scientific community and did not explore those of the lay public in any depth.

### Recommendations and further research

We think the COVID-19 pandemic is the perfect time for research into new strategies to prevent or reduce misinformation. There is huge public interest in scientific discovery and the need for adequate funding, and widespread awareness about the problem of misinformation.^1^ At the same time, the public have received a crash course in science and information literacy, with growing understanding of important concepts like p values, peer review, systematic reviews and uncertainty.^77^ Building on this new knowledge could promote improvements in public understanding of the scientific process and how to find reliable science news items. We recommend ongoing research into specific policies and practical tools aimed at reducing misinformation such as those generated by our findings from participants’ insights and expertise. The broader exploration of how and why people respond to new science information about topics such as the COVID-19 pandemic will be another important area of global research.^75 78 79^ The pandemic has generated a rich set of data about this topic that can be analysed in detail for many years to come.

## Conclusions

Misinformation about COVID-19 is a problem because a pandemic relies on rapid mass behavioural change to minimise harm to global health. Our results show widespread concern from within the scientific community about systemic failings that might facilitate the production and spread of false or misleading science information. We advocate for further research into practical ways to prevent misinformation that target multiple points along the science production and communication pathway. We need to work together to minimise the production and spread of misinformation about COVID-19 and other science crises in the future.

## Data Availability

No data are available. In order to protect participant confidentiality, we are not able to provide interview transcripts.
